# Supplementary material for: Software evaluation for de novo detection of transposons
Source: Mob DNA. 2022 Apr 27;13:14. doi: 10.1186/s13100-022-00266-2 (PMC9047281; doi:10.1186/s13100-022-00266-2)
Supplement: Supplementary file 1 — Additional file 1. [file 13100_2022_266_MOESM1_ESM.pdf]

Supplementary materials

Table S1. TEs inserted into simulated data. Transposon ids after Dfam. These TEs were inserted at random positions into a random sequence with 42% GC content.

TE_id	Count	Average identity	Standard deviation	Number of indels	Target site duplication	Length	Number of truncated elements	Number of nested element
**AluY**	730	87	12	16	y	311	67	11
**BEL12-I_DR**	550	89	12	10	y	7730	80	12
**Copia1_DM**	680	87	14	10	y	4530	65	10
**DIRS-1_DR**	710	85	15	12	n	5634	70	5
**G5_DM-Jockey**	590	91	10	15	y	4856	75	16
**GYPSY8_DM**	470	84	10	15	y	4955	74	12
**hAT-9_DR**	480	82	12	11	y	2386	65	13
**Helitron-1N3_DR**	550	83	10	11	n	1843	65	10
**HERVL**	590	88	14	14	y	6542	50	7
**L1-4_DR**	620	78	9	16	y	5548	72	13
**Merlin1_HS**	580	80	11	13	y	1175	60	18
**Ngaro1_DR**	560	86	12	11	n	6578	73	9
**Penelope-1_DR**	810	85	11	20	y	4446	60	11
**piggyBac-N1_DR**	610	80	10	19	y	1005	64	20
**Polinton-1_DR**	420	81	11	20	n	18485	70	10
**RTE1**	570	90	13	18	y	3291	71	11
**SINE3-1**	580	85	13	10	y	590	70	7
**TC1-2_DM**	590	83	14	12	y	1644	70	16
**TRANSIB1**	670	81	10	12	y	3014	68	9
**Turmoil2**	650	79	9	10	y	6999	69	15

Table S2. Comparison of the consensus models obtained for each single TE inserted in the simulated sequence. With * are indicated models were the models include a duplication or an extension of the same TE and with # are indicated models that are the combination of two TEs which originated from nested TEs.

Original TE-families		Length of the models created		
TE	Length	RepeatScout	RepeatModeler	REPET
AluY	311	308	345<br>320<br>310	-
BEL12	7730	7724	7751<br>7730<br>6413	11148*<br>7750<br>2769
Copia	4530	8843*	4532<br>4514<br>4326<br>4076	4652<br>4314
DIRS	5654	5644<br>9385*	5672<br>5653<br>5610<br>5445<br>4633<br>4062	7134#<br>5401<br>1824<br>1727
G5	4856	4854	4851<br>4843<br>4836<br>3151	7502 *<br>5232<br>5197<br>4904<br>3967<br>2359
GYPSY	4955	4360<br>3920	4938<br>4012<br>3951<br>3373	5068<br>4682<br>2061<br>1445<br>1256
hAT	2386	2380	2380<br>2380	2426
Helitron	1843	1839<br>1472	1832<br>1823<br>1234	1907<br>1906<br>1744<br>871<br>633

Original TE-families		Length of the models created		
**TE**	**Length**	**RepeatScout**	**RepeatModeler**	**REPET**
HERVL	6542	12634*	6549<br>6523<br>6094	11495#<br>6553<br>3886<br>3169
L1	5548	4460<br>3379<br>2415	5496<br>5488<br>3386<br>2588<br>2478<br>1289	5784<br>5605<br>5443<br>2855<br>2142<br>1794<br>1639<br>1179<br>667
Merlin	1175	1168	1172<br>1167<br>744	1231<br>1216<br>1089<br>629
Ngaro	6578	6571<br>6020	6568<br>6566<br>6563<br>6156	7798#<br>6652<br>6379<br>2487<br>2445
Penelope	4446	4437<br>2276	4432<br>4407<br>4189<br>3910<br>3198<br>3908	5077<br>3251<br>812
piggyBac	1005	997	1002<br>990<br>840	1067<br>1055<br>966<br>641<br>477
Polinton	18485	14288<br>10127<br>4536<br>1513	17626<br>13482<br>11066<br>9356<br>8330<br>6655<br>372<br>169	16912<br>16015<br>15368<br>10418<br>6631<br>2793<br>1690<br>1216

Original TE-families		Length of the models created		
**TE**	**Length**	**RepeatScout**	**RepeatModeler**	**REPET**
RTE1	3291	3287	3286<br>3270<br>3212	3343<br>3200<br>3128<br>1222
SINE3	590	587	587<br>581<br>578	624<br>559
TC1	1644	1634	1636<br>1627<br>1472	1689<br>771
TRANSIB	3014	3002	2995<br>2546<br>2378	3094<br>2858<br>1166
Turmoil	6999	6676	7016<br>6985<br>6974<br>6970<br>4201	7113<br>6598<br>5956<br>5834#<br>1757<br>1381<br>1100

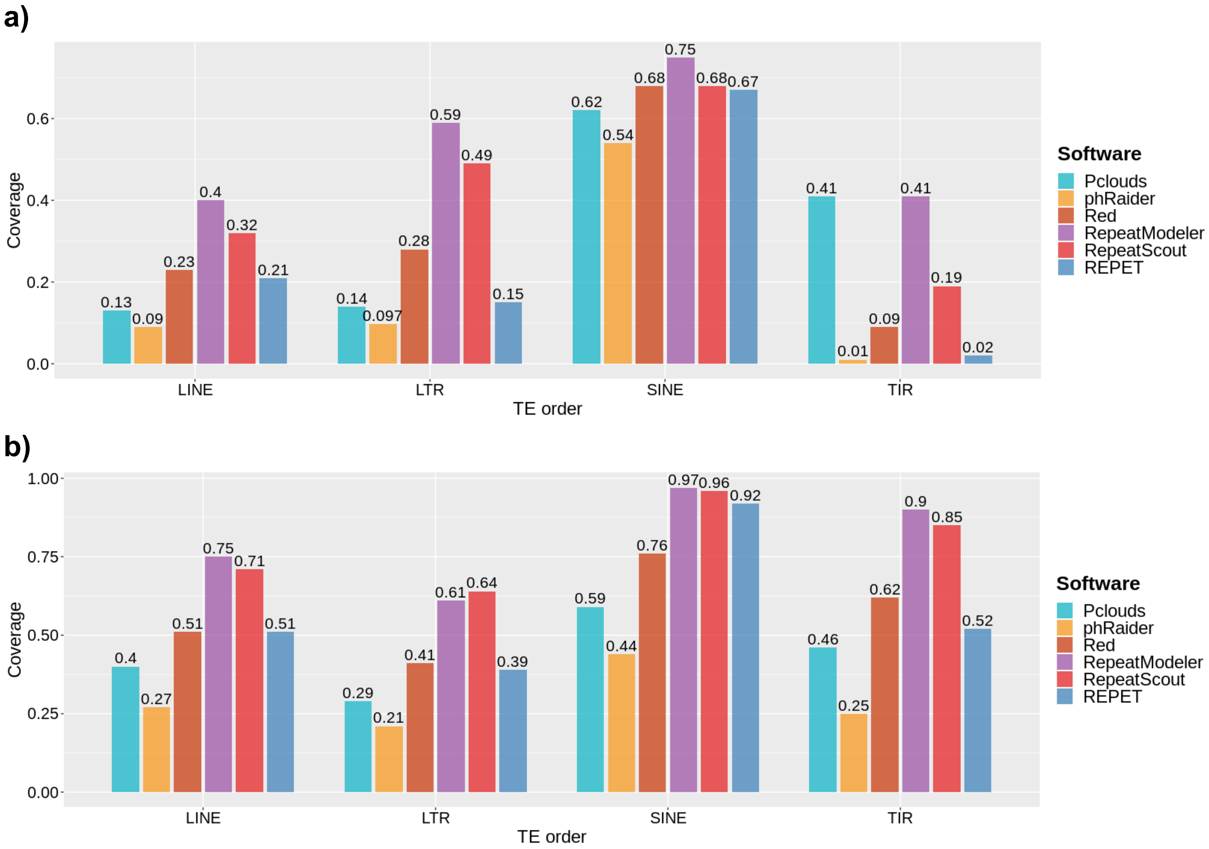

Figure S1. Coverage of each TE order in the human (a) and zebrafish (b) dataset.
